# Supplementary material for: Empirical methods for controlling false positives and estimating confidence in ChIP-Seq peaks
Source: BMC Bioinformatics. 2008 Dec 5;9:523. doi: 10.1186/1471-2105-9-523 (PMC2628906; doi:10.1186/1471-2105-9-523)
Supplement: Additional File 1 — A variety of html documents from the USeq web site detailing the available applications, their best usage, output file type descriptions, command line menus, etc. [file 1471-2105-9-523-S1.zip › USeqUserGuides/JavaDoc.zip!/JavaDoc/expr/CorrelationMaps.html]

CorrelationMaps


|  |  |  |
| --- | --- | --- |
| **Overview** \| **Package** \| **Class** \| **Use** \| **Tree** \| **Deprecated** \| **Index** \| **Help** | |  |
| PREV CLASS   **NEXT CLASS** | **FRAMES**    **NO FRAMES**     **All Classes** |  |
| SUMMARY: NESTED \| FIELD \| CONSTR \| METHOD | DETAIL: FIELD \| CONSTR \| METHOD |  |


---


## expr Class CorrelationMaps

```
java.lang.Object
  expr.CorrelationMaps
```

---

``` public class CorrelationMaps extends java.lang.Object ```

---

| **Field Summary** | |
| --- | --- |
| `static float[]` | `SQUARE_ROOTS` |


**Constructor Summary**

`CorrelationMaps(java.lang.String[] args)`


| **Method Summary** | |
| --- | --- |
| `void` | `assignPValues()`             Assigns -10Log10(pvalues) for each score > 0 in the CorrelationWindows |
| `float` | `averageScores(ExpressedGene[] ex)`             Average scores \* square root of number of genes. |
| `void` | `correlate()`             Creates CorrelationWindows and performs random trials. |
| `void` | `filterGenes()` |
| `static void` | `main(java.lang.String[] args)` |
| `ExpressedGene[]` | `makeRankedFloatArrays(ExpressedGene[] ex)`             Makes and places RankedFloatArrays in each ExpressedGene, also nulls the ExpressedGene.values. |
| `float[]` | `minMax()` |
| `float` | `pairwiseCorrelationScore(ExpressedGene[] ex)`             Average coorelation coefficient \* square root of number of genes. |
| `static ExpressedGene[]` | `parseGeneFile(java.io.File geneFile)`             Converts a tab delimited file of (name chr start stop val1 val2 ...) into an ExpressedGene[] |
| `void` | `printBarFiles()` |
| `void` | `printCorrelationWindows()`             Prints a spread sheet summary and a xxx.egr file for all of the windows. |
| `static void` | `printDocs()` |
| `void` | `processArgs(java.lang.String[] args)`             This method will process each argument and assign new varibles |
| `void` | `randomize(int index)` |
| `ExpressedGene[][]` | `window(ExpressedGene[] ex)`             Splits a chromosome of ExpressedGenes into windows based on the windowSize and minNumberGenes |

**Methods inherited from class java.lang.Object**

`equals, getClass, hashCode, notify, notifyAll, toString, wait, wait, wait`

**Field Detail**

### SQUARE\_ROOTS

```
public static final float[] SQUARE_ROOTS
```


**Constructor Detail**

### CorrelationMaps

```
public CorrelationMaps(java.lang.String[] args)
```


**Method Detail**

### filterGenes

```
public void filterGenes()
```

---


### printBarFiles

```
public void printBarFiles()
```

---


### printCorrelationWindows

```
public void printCorrelationWindows()
```

:   Prints a spread sheet summary and a xxx.egr file for all of the windows.

---


### assignPValues

```
public void assignPValues()
```

:   Assigns -10Log10(pvalues) for each score > 0 in the CorrelationWindows

---


### minMax

```
public float[] minMax()
```

---


### correlate

```
public void correlate()
```

:   Creates CorrelationWindows and performs random trials.
    Nulls various unused objects.

---


### randomize

```
public void randomize(int index)
```

---


### pairwiseCorrelationScore

```
public float pairwiseCorrelationScore(ExpressedGene[] ex)
```

:   Average coorelation coefficient \* square root of number of genes.

---


### averageScores

```
public float averageScores(ExpressedGene[] ex)
```

:   Average scores \* square root of number of genes.

---


### window

```
public ExpressedGene[][] window(ExpressedGene[] ex)
```

:   Splits a chromosome of ExpressedGenes into windows based on the windowSize and minNumberGenes

---


### makeRankedFloatArrays

```
public ExpressedGene[] makeRankedFloatArrays(ExpressedGene[] ex)
```

:   Makes and places RankedFloatArrays in each ExpressedGene,
    also nulls the ExpressedGene.values.

---


### parseGeneFile

```
public static ExpressedGene[] parseGeneFile(java.io.File geneFile)
```

:   Converts a tab delimited file of (name chr start stop val1 val2 ...) into an ExpressedGene[]

---


### main

```
public static void main(java.lang.String[] args)
```

---


### processArgs

```
public void processArgs(java.lang.String[] args)
```

:   This method will process each argument and assign new varibles

---


### printDocs

```
public static void printDocs()
```


---


|  |  |  |
| --- | --- | --- |
| **Overview** \| **Package** \| **Class** \| **Use** \| **Tree** \| **Deprecated** \| **Index** \| **Help** | |  |
| PREV CLASS   **NEXT CLASS** | **FRAMES**    **NO FRAMES**     **All Classes** |  |
| SUMMARY: NESTED \| FIELD \| CONSTR \| METHOD | DETAIL: FIELD \| CONSTR \| METHOD |  |


---
